# Cylindroma with Stromal Adipose Tissue Metaplasia versus Arising in a Background of Nevus Lipomatosus

**DOI:** 10.1155/2014/203298

**Published:** 2014-03-16

**Authors:** Ryan Yu, Salem Alowami

**Affiliations:** ^1^Department of Pathology and Molecular Medicine, McMaster University, HSC-2N10, 1280 Main Street West, Hamilton, ON, Canada L8S 4K1; ^2^St. Joseph's Healthcare Hamilton, Charlton Campus, 50 Charlton Avenue East, Hamilton, ON, Canada L8N 4A6

## Abstract

Nevus lipomatosus superficialis is a rare type of connective tissue nevus. Cylindroma is a benign skin appendage tumor with a predilection for the scalp of older females. We describe the case of a 56-year-old woman with a scalp lesion demonstrating histopathologic features consistent with benign cylindroma arising within a nevus lipomatosus superficialis. To our knowledge, this lesion has not been raised in the literature in the differential for cylindroma with what is presumed to be stromal adipose metaplasia.

## 1. Introduction

Nevus lipomatosus superficialis (NLS) is a rare type of connective tissue nevus that was first described in 1921 by Hoffmann and Zurhelle [1]. It is characterized by the ectopic presence of mature adipose tissue in the dermis. Clinically, two variants of NLS are recognized: classic and solitary. The classic variant of NLS is present in the first two decades of life as aggregations of flesh-colored or yellow papules and nodules with a predilection for the buttocks, upper posterior thighs, and lumbar back. The solitary variant of NLS is present in adults, predominantly after the third decade of life, as isolated papules or nodules anywhere on the skin. Compared with the classic variant of NLS, the solitary variant shows more frequent involvement of non-pelvic-girdle areas of skin, including the axilla, arm, knee, ear, and scalp [2]. Unlike nevus sebaceus of Jadassohn, it is rare for appendageal tumors to develop within NLS. Cylindroma, in particular, is a benign skin appendage tumor of hair follicle origin [3] with a predilection for the scalp of middle-aged to elderly females. We present the case of a patient with a scalp lesion demonstrating histopathologic features consistent with benign cylindroma arising within a nevus lipomatosus superficialis.

## 2. Case Presentation

**Figure 1:**
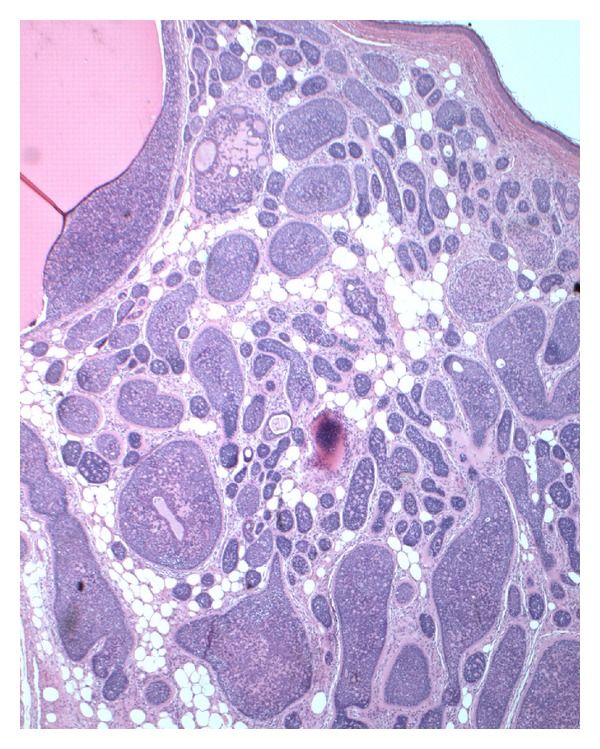
Dermally located basaloid nests with surrounding eosinophilic bands. Adipocytes are present in the stroma extending to the papillary dermis (H&E, 20x).

**Figure 2:**
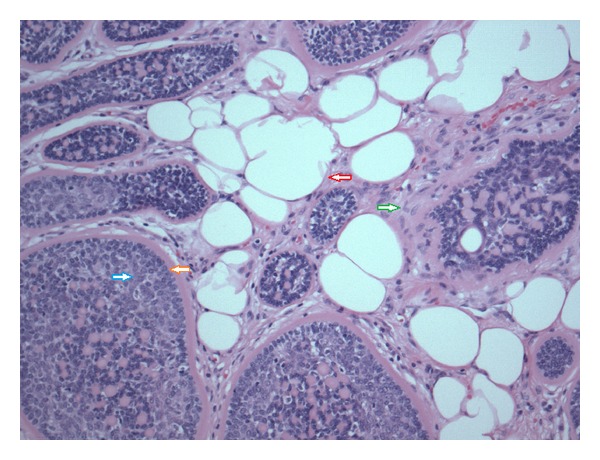
High power view of cylindroma and surrounding adipocytes. Red arrow: adipocyte. Green arrow: collagenous stroma. Orange arrow: peripheral cell with dark nucleus. Blue arrow: more centrally located cell with pale nucleus (H&E, 100x).

**Figure 3:**
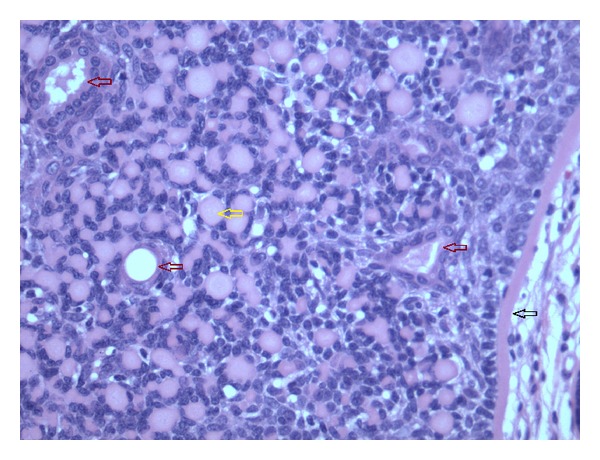
High power view of cylindroma. Black arrow: hyaline sheath. Maroon arrows (3): duct-like structures, two with intraluminal amorphous eosinophilic content. Yellow arrow: hyaline globule (H&E, 200x).

A 56-year-old woman presented with a 1.1 cm skin-colored nodule on the right anterior scalp at the hairline. The remaining physical examination was noncontributory. Her medical history included chronic obstructive pulmonary disease and hypothyroidism. An excisional biopsy was performed. Microscopic examination showed a poorly circumscribed tumor with variably-sized, irregularly-shaped islands of basaloid cells ensheathed in bright eosinophilic bands (Figures 1 and 2). The islands occupied the dermis and extended into the subcutaneous fat to the depth of fascia. A band of compressed papillary dermis separated the basaloid islands from the overlying epidermis. The tumor islands were dispersed by an expanded stroma containing adipocytes distributed circumferentially around, but not within, the islands as well as scattered groups and single cells to the level of the papillary dermis. The islands at the peripheral extent of the tumor were flanked by a narrow vertical rim of adipocytes before abrupt transition to normal dermis. Most of the islands were composed peripherally of cells with small, dark-staining nuclei. Cells with larger, paler nuclei were found more centrally. No nuclear pleomorphism or mitotic figures were identified. Most of the islands contained small eosinophilic globules and duct-like structures, some of which enclosed faintly eosinophilic, amorphous material (Figure 3). Histochemistry showed the eosinophilic bands and globules were periodic acid-Schiff- (PAS-) positive and diastase-resistant. Immunostain for S-100 protein highlighted the presence of dendritic cells within the tumor islands. The findings were consistent with a cylindroma arising within a nevus lipomatosus superficialis.

## 3. Discussion

Nevus lipomatosus superficialis is a hamartomatous lesion of uncertain pathogenesis. The ectopic adipose cells are thought by some authors to originate from vascular elements in a process likened to fetal adipogenesis [4, 5]. The occurrence of other lesions in NLS has been reported, albeit rarely, predominantly as pilar abnormalities. Lee et al. [6] described cystically dilated hair follicles with infundibular keratinization lining. Similarly, Yun et al. [7] described dilated follicular cysts, as well as hair follicles that formed anastomosing epithelial strands suggestive of fibrofolliculoma. Sakanoue et al. [8] reported a case of NLS associated with a cystic follicular structure accompanied by sebaceous glands embedded in fibrous stroma, compatible with trichofolliculoma. Bancalari et al. [9] described two cases with features of folliculosebaceous cystic hamartoma, which may be regarded as the late stage of trichofolliculoma [10]. To our knowledge, cylindroma has not been previously described in association with NLS. The explanation rests in the diagnostic challenge of differentiating a cylindroma arising within a nevus lipomatosus superficialis from a cylindroma with what is presumed to be stromal adipose metaplasia. In the latter, adipocytes are generally dispersed throughout the lesion as solitary units or small clusters [11]. While the present case might represent a rare example of extensive adipose metaplasia affecting most of the stroma, the presence of the adipose tissue around the cylindroma tumor nests circumferentially and the presence of the cylindroma almost buried in adipose tissue, rather than few scattered adipocytes within the stroma as typical of metaplasia, make us favor cylindroma arising in a background of nevus lipomatosus. From the perspective of management, the distinction is largely academic. Because both diagnoses are benign, complete surgical excision should be sufficient for treatment, which may not be necessary other than for cosmesis.
